# The coexistence of carotid and lower extremity atherosclerosis further increases cardio-cerebrovascular risk in type 2 diabetes

**DOI:** 10.1186/s12933-016-0360-2

**Published:** 2016-03-05

**Authors:** Mei-Fang Li, Cui-Chun Zhao, Ting-Ting Li, Yin-Fang Tu, Jun-Xi Lu, Rong Zhang, Ming-Yun Chen, Yu-Qian Bao, Lian-Xi Li, Wei-Ping Jia

**Affiliations:** Department of Endocrinology and Metabolism, Shanghai Diabetes Institute, Shanghai Clinical Center for Diabetes, Shanghai Key Clinical Center for Metabolic Diseases, Shanghai Key Laboratory of Diabetes Mellitus, Shanghai Jiao Tong University Affiliated Sixth People’s Hospital, 600 Yishan Road, Shanghai, 200233 China; Department of Emergency, Shanghai Jiao Tong University Affiliated Sixth People’s Hospital, 600 Yishan Road, Shanghai, 200233 China; Department of VIP, Shanghai Jiao Tong University Affiliated Sixth People’s Hospital, 600 Yishan Road, Shanghai, 200233 China

**Keywords:** Type 2 diabetes, Carotid atherosclerosis, Lower limb atherosclerosis, Cardio-cerebrovascular events, Self-reported cardio-cerebrovascular diseases

## Abstract

**Background:**

Both carotid and lower limb atherosclerosis are associated with increased cardiovascular and cerebrovascular risks. However, it is still unclear whether the concomitant presence of carotid and lower extremity atherosclerosis further increases the cardiovascular and cerebrovascular risks. Therefore, our aim is to investigate whether the coexistence of carotid and lower extremity atherosclerosis was associated with higher cardiovascular and cerebrovascular risks in patients with type 2 diabetes.

**Methods:**

This cross-sectional study was performed in 2830 hospitalized patients with type 2 diabetes. Based on carotid and lower limb Doppler ultrasound results, the patients were divided into three groups including 711 subjects without atherosclerosis, 999 subjects with either carotid or lower limb atherosclerosis, and 1120 subjects with both carotid and lower limb atherosclerosis. And we compared the clinical characteristics and prevalence of both cardio-cerebrovascular events (CCBVEs) and self-reported cardio- cerebrovascular diseases (CCBVDs) among the three groups.

**Results:**

After adjusting for age, sex, and duration of diabetes, there were significant increases in the prevalence of both CCBVEs (3.8 vs. 11.8 vs. 26.4 %, p < 0.001 for trend) and self-reported CCBVDs (6.9 vs. 19.9 vs. 36.5 %, p < 0.001 for trend) across the three groups (diabetics without atherosclerosis, diabetics with either carotid or lower limb atherosclerosis, and diabetics with both carotid and lower extremity atherosclerosis). A fully adjusted logistic regression analysis also revealed that compared with those without atherosclerosis, those with either carotid or lower limb atherosclerosis had higher risk of CCBVEs (OR 1.724, 95 % CI 1.001–2.966) and self-reported CCBVDs (OR 1.705, 95 % CI 1.115–2.605), and those with concomitant presence of carotid and lower extremity atherosclerosis had the highest risk of CCBVEs (OR 2.869, 95 % CI 1.660–4.960) and self-reported CCBVDs (2.147, 95 % CI 1.388–3.320)(p < 0.001 for trend in CCBVEs and p = 0.002 for trend in CCBVDs, respectively).

**Conclusions:**

Either carotid or lower limb atherosclerosis was obviously related to increased cardio-cerebrovascular risk in type 2 diabetes. The concomitant presence of carotid and lower extremity atherosclerosis further increased cardio-cerebrovascular risk in patients with type 2 diabetes. The combined application of carotid and lower extremity ultrasonography may help identify type 2 diabetics with higher cardio-cerebrovascular risk.

## Background

Epidemiological and clinical studies frequently indicate that people with type 2 diabetes have elevated risk to get a number of serious atherosclerotic vascular problems, especially cardio-cerebrovascular diseases (CCBVDs) [1–5]. For example, a prospective observational study displayed that each 1 % increase in the glycated hemoglobin level is associated with an increase of 14 % in the risk of myocardial infarction [6]. Moreover, in the Multiple Risk Factor Intervention Trial of 347,978 men, diabetic men were three times more likely to develop a stroke than nondiabetic ones [7]. Therefore, early identification of subjects with high cardio-cerebrovascular risk is quite crucial in type 2 diabetes.

Several studies have demonstrated that indices such as systemic atherosclerosis, cardio-ankle vascular index, ankle brachial index and pulse wave velocity are closely related to cardiovascular risk in diabetic populations [8–12]. However, accurate measurement of above index requires specialized training and demands a tedious and long procedure, and thus they probably could not be widely used in routine clinical practice especially remote areas in China.

Numerous Studies have demonstrated that artery intima-media thickness (IMT) and plaques in carotid, femoral are associated with increased risk of cardio-cerebrovascular events (CCBVEs) and CCBVDs [13–19]. For example, the Osaka Follow-Up Study demonstrated that every 1-SD increment in carotid IMT was obviously associated with a 1.57 hazard ratio in the cardiovascular events [20]. And most importantly, two prospective data indicated that evaluations of plaques provided better prediction for incident cardiovascular events than assessments of IMT in patients with stable angina and patients with end-stage renal disease [21, 22]. Collado et al. [23] recently also reported that the presence of carotid plaque, especially, calcified plaque, were predictors of new cardiovascular events and cardiovascular mortality, but carotid IMT was not in hemodialysis patients.

However, in some studies, no significant relationship was observed between IMT, plaque and CCBVEs, CCBVDs after adjusting for other vascular risk factors [24, 25]. For example, during Seven-Year Follow-Up, Yuk et al. [25] found that coronary artery disease patients with either carotid plaque or thick IMT had higher incidence and prediction rates of stroke, but this was not statistically significant after being adjusted in multivariate analysis. Furthermore, in comparison with carotid and femoral plaques, very few studies are available regarding the correlation between the concomitant carotid and lower limb atherosclerosis and CCBVEs and CCBVDs.

Therefore, the purposes of our study were to determine whether the presence of either carotid or lower limb atherosclerosis increases cardiovascular and cerebrovascular risks indicated by CCBVEs and self-reported CCBVDs, and further to verify whether the concomitant presence of carotid and lower extremity atherosclerosis further increases the cardio-cerebrovascular risk independent of usual cardiovascular risk factors in patients with type 2 diabetes.

## Methods

### Subjects and study design

Our present study was cross-sectional and the data partly came from our previous studies [26–33]. That is, 3598 patients with type 2 diabetes mellitus hospitalized for either comprehensive diabetic complications screening or poor blood glucose control in our department were continuously observed from January 2007 to June 2009. And 768 subjects were eliminated as the following reasons: incomplete physical examination and clinical parameters, and lack of carotid or lower limb ultrasound examinations. Ultimately, 2830 participants joined in our following analysis, including 1246 males and 1584 females.

We recorded all participants’ information, such as duration of diabetes (DD), smoking and drinking habits, the history of hypertension, the administered drugs including insulin or insulin analogues (IIAs), lipid-lowering drugs (LLDs), antihypertensive agents (AHAs) and anti-platelet agents (APAs), cardiovascular events (CVEs), cerebrovascular events (CBVEs), CCBVEs, self-reported cardiovascular diseases (CVDs), self-reported cerebrovascular diseases (CBVDs), and self-reported CCBVDs. Our current study was approved by the ethics committee of Shanghai Jiao Tong University Affiliated Sixth People’s Hospital, and written informed consent was obtained from all participants.

### Physical and laboratory examinations

The physical and laboratory examinations used in our present study have been clearly described in our previous studies [26–33]. Briefly, height, weight, waist circumference, hip circumference, and blood pressure were measured according to standard protocols. The body mass index (BMI) and the waist-to-hip ratio (WHR) were based on the corresponding formula to calculate [26]. Blood samplings were performed to test total cholesterol (TC), total triglycerides (TTG), high-density lipoprotein cholesterol (HDL-C), low-density lipoprotein cholesterol (LDL-C), glycosylated hemoglobin A1C (HbA1c), fasting plasma glucose(FPG), 2-h postprandial plasma glucose (2h PPG), fasting C-peptide(FCP), 2-h postprandial C-peptide (2h PCP), alanine aminotransferase (ALT), creatinine (Cr), serum uric acid (SUA), C-reactive protein(CRP). The 24-h urinary albumin excretion (UAE) was calculated as the mean from three separate urine samples. The estimated glomerular filtration rate (eGFR) was calculated using the simplified MDRD formula: eGFR = 186.3 × (Serum creatinine)^−1.154^ × (age)^−0.203^ (×0.742 if female).

### Ultrasonography examinations

Doppler ultrasonography examinations of carotid and lower limb arteries, involving in the measurement of intima-media thickness, atherosclerotic plaque and stenosis, have been previously described in details [26, 27, 30, 32]. That is, the ultrasonographic examination was carried out by three experienced ultrasonographers using a machine Acuson Sequoia 512 with a probe of 5–13-MHz MHz according to a standardized protocol. After the participants had kept in the supine position for 5 min, the transducer was successively placed on the neck and lower limbs to manifest blood vessel imaging and blood flow characteristics. At each location, IMT and atherosclerotic plaques were recorded. Carotid arteries were examined bilaterally at the levels of the common carotid arteries, the bifurcation, the external carotid arteries, and the internal carotid arteries from transverse and longitudinal orientations. Lower limb arteries were evaluated bilaterally at the levels of the seven locations: common femoral artery, profunda femoris artery, superficial femoral artery, popliteal artery, anterior tibial artery, posterior tibial artery and peroneal artery. The measurement reproducibilities of the above atherosclerotic lesions have also been indicated previously [27, 32].

### Diagnostic criteria

Dyslipidemia was defined as TG ≥ 1.7 mmol/L, or LDL-C ≥ 3.37 mmol/L, or TC ≥ 5.18 mmol/L, or HDL-C < 1.04 mmol/L, or use of LLDs. The definitions of CVEs, CBVEs, CCBVEs, carotid and lower extremity atherosclerosis were carried out referring to our previous studies [26, 28, 31]. Briefly, CVEs were defined based on a history of myocardial infarction, angina, angioplasty, or coronary artery bypass surgery. CBVEs were defined based on a history of transient ischemic attack, ischemic, or hemorrhagic stroke. CCBVEs were defined based on histories of CVEs and/or CBVEs. According to the Mannheim consensus [34], atherosclerotic plaques were defined as focal structures encroaching into the arterial lumen of 0.5 mm or 50 % of the surrounding IMT value or IMT of >1.5 mm. The definitions of carotid and lower limb arteriosclerosis were based on our previous studies [26, 27]. Briefly, carotid atherosclerosis was defined as the presence of carotid arterial atherosclerotic plaque in any of bilateral carotid artery segments including the common, the bifurcation, the external, and the internal carotid arteries. Lower extremity atherosclerosis was defined as the presence of lower extremity arterial atherosclerotic plaque in any of bilateral lower extremity artery segments including the common, the profunda and the superficial femoral arteries, and popliteal arteries, anterior tibial arteries, posterior tibial arteries, and peroneal arteries.

Self reported CVDs were defined as reporting of one or more of the following conditions: coronary artery disease, congestive heart failure, or CVEs. Self reported CBVDs were defined as reporting of one or more of the following conditions: radiologic findings of cerebrovascular diseases or CBVEs. Self-reported CCBVDs were defined as having self-reported CVDs and/or CBVDs.

### Statistical analysis

SPSS 15.0 was performed to analyze the data. Data were represented as either mean ± SD or median or percentage. One-way ANOVA with LSD was used to calculate group comparisons for continuous variables with normal distribution. Kruskal–Wallis test was conducted for continuous variables with non-normal distribution. Chi squared statistical analysis was used for comparisons in categorical variables. Binary multiple regression analysis was conducted to identify the correlations between the concomitant presence of carotid and lower extremity atherosclerosis and CCBVEs, self-reported CCBVDs. p < 0.05 (two-sided) was considered as statistical significance.

## Results

### Clinical and biochemical characteristics of the study subjects

The patients were divided into three groups including subjects without atherosclerosis, with either carotid or lower limb atherosclerosis, and with concomitant carotid and lower limb atherosclerosis. Table 1 displays the clinical and laboratory characteristics of the studied subjects among the three groups. Age, sex, DD, alcohol, prevalence of hypertension and dyslipidemia, proportion of use of IIAs, LLDs, AHAs and APAs, systolic blood pressure (SBP), diastolic blood pressure (DBP), TC, LDL-C, 2h C-P, Cr, UAE and SUA were significantly different among the three groups when age and sex were examined (all p < 0.05).

**Table 1:** Characteristics of the study subjects

Variables	Without AS (n = 711)	With either carotid or lower limb AS (n = 999)	With both carotid and lower limb AS (n = 1120)	p value	*p value
Age (months)	49 ± 11	60 ± 10	67 ± 10	<0.001	<0.001
Male (n, %)	325 (45.7 %)	446 (44.6 %)	475 (42.4 %)	0.340	<0.001
DD (months)^a^	48 (3–96)	84 (24–132)	120 (48–168)	<0.001	<0.001
Smoking (n, %)	226 (31.79 %)	291 (29.13 %)	281 (25.09 %)	0.007	0.612
Alcohol (n, %)	115 (16.17 %)	171 (17.12 %)	142 (12.68 %)	0.014	0.036
Hypertension (n, %)	256 (36.00 %)	521 (52.20 %)	742 (66.20 %)	<0.001	<0.001
Dyslipidemia (n, %)	546 (76.8 %)	729 (73.0 %)	857 (76.5 %)	0.041	0.001
IIAs (n, %)	486 (68.4 %)	687 (68.8 %)	849 (75.8 %)	<0.001	<0.001
LLD (n, %)	189 (26.6 %)	292 (29.2 %)	402 (35.9 %)	<0.001	<0.001
AHAs (n, %)	224 (31.5 %)	479 (47.9 %)	692 (61.8 %)	<0.001	<0.001
APAs (n, %)	96 (13.5 %)	534 (53.5 %)	827 (73.8 %)	<0.001	<0.001
BMI (kg/m2)	24.97 ± 3.48	24.92 ± 3.41	24.78 ± 3.43	0.476	0.888
WHR (cm)	0.91 ± 0.06	0.91 ± 0.06	0.92 ± 0.07	0.004	0.206
SBP (mmHg)	126 ± 15	132 ± 17	136 ± 18	<0.001	<0.001
DBP (mmHg)	80 ± 10	80 ± 10	80 ± 10	0.448	0.006
TC (mmol/l)	4.72 ± 1.14	4.72 ± 1.13	4.74 ± 1.15	0.943	<0.001
TTG (mmol/l)^a^	1.57 (1–2.48)	1.39 (0.97–2.12)	1.39 (1–1.96)	<0.001	0.456
HDL-C (mmol/l)	1.09 ± 0.32	1.13 ± 0.3	1.12 ± 0.3	0.018	0.120
LDL-C (mmol/l)	3.07 ± 0.94	3.09 ± 0.9	3.15 ± 1.01	0.148	<0.001
HbA1C (%)	9.22 ± 2.36	9.05 ± 2.36	9.12 ± 2.46	0.346	0.290
FPG (mmol/l)^a^	8.05 (6.56–10.09)	7.61 (6.17–9.67)	7.55 (6.01–9.39)	<0.001	0.572
2h PPG (mmol/l)^a^	13.76 (10.32–17.18)	13.23 (9.94–16.49)	13.66 (10.22–16.98)	0.246	0.519
FCP^a^	1.69 (1.03–2.48)	1.68 (1.07–2.44)	1.61 (0.92–2.43)	0.223	0.234
2h PCP^a^	3.72 (2.17–5.39)	3.81 (2.22–5.47)	3.49 (1.92–5.26)	0.034	0.002
ALT (U/L)^a^	21 (15–35)	20 (14–31)	17 (12–26)	<0.001	0.225
Cr (mmol/l)^a^	62 (51–73)	67 (56–79)	71 (58–87)	<0.001	0.003
UAE (mg/24 h)^a^	9.8 (6.2–20.1)	11.06 (6.3–25.88)	12.4 (7.1–50)	<0.001	0.005
eGFR (ml/min/1.73 m^2^) m^2^)	113 (98–132)	99 (84–118)	92 (74–111)	<0.001	0.370
SUA (μmol/l)^a^	308 (252–366)	305 (254–367)	317 (260–383)	0.003	0.034
CRP^a^	0.99 (0.43–2.26)	1.07 (0.46–2.52)	1.36 (0.6–3.8)	<0.001	0.198

Values are presented as mean ± SD, median with interquartile range, or percentages

^a^Non-normal distribution of continuous variables

p value: The p values were not adjusted for age and sex for the trend

* p value: The * p value were adjusted by sex and age for the trend

### Characteristics of CCBVEs and self-reported CCBVDs of the whole subjects

**Fig. 1:**
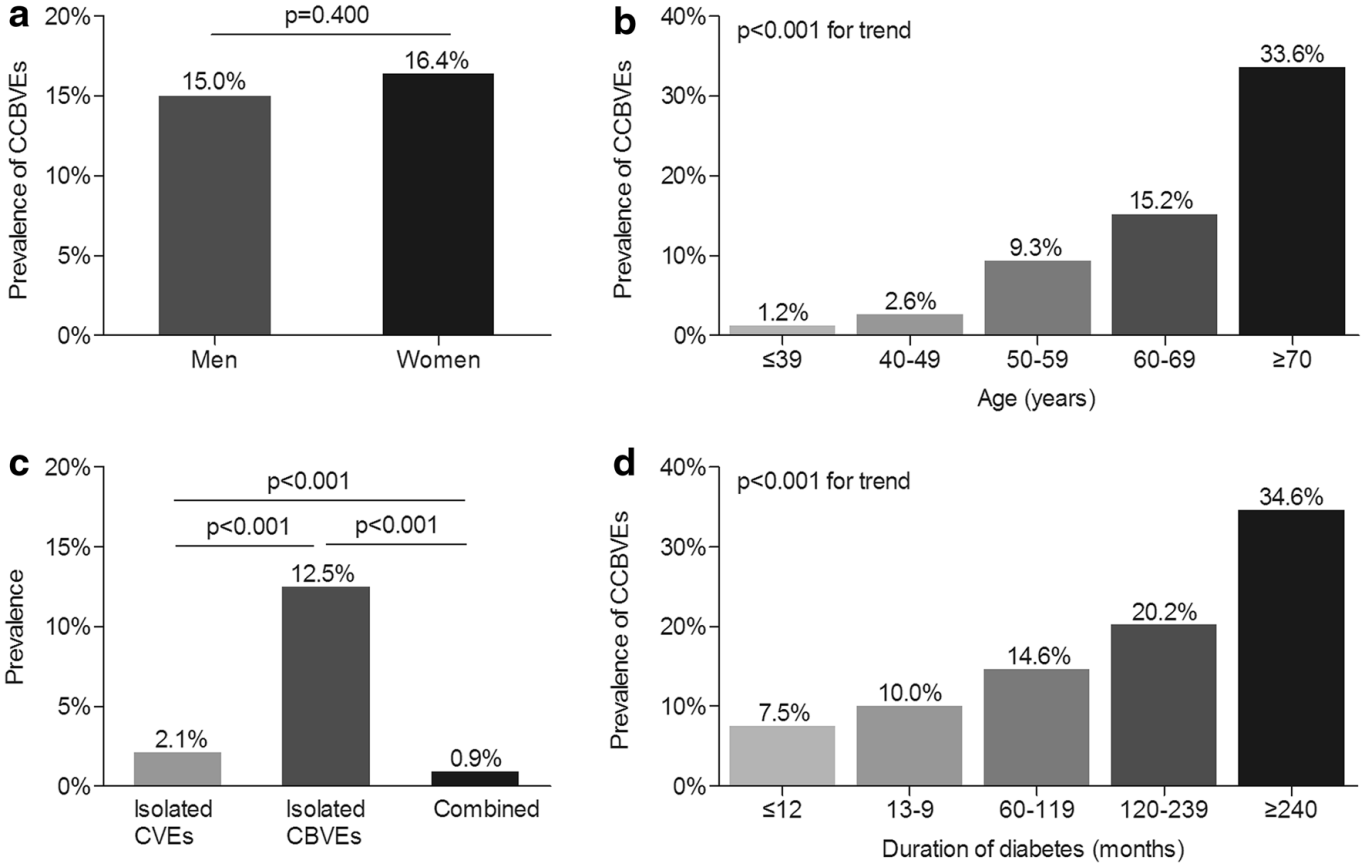
Characteristics of CCBVEs of the entire subjects **a** The prevalence of CCBVEs stratified by sex after adjusting on age and DD. The p value for sex comparison was 0.400. **b** The prevalence of CCBVEs stratified by age after adjusting on sex and DD. The p value for group comparison was <0.001. **c** The distribution of CCBVEs after adjusting on sex, age and DD. The prevalence of isolated CVEs, isolated CBVEs and combined CVEs and CBVEs was successively 2.1, 12.5 and 0.9 %. The p value for group comparison was <0.001. **d** The prevalence of CCBVEs stratified by duration of diabetes after adjusting on sex and age. The p value for group comparison was <0.001

**Fig. 2:**
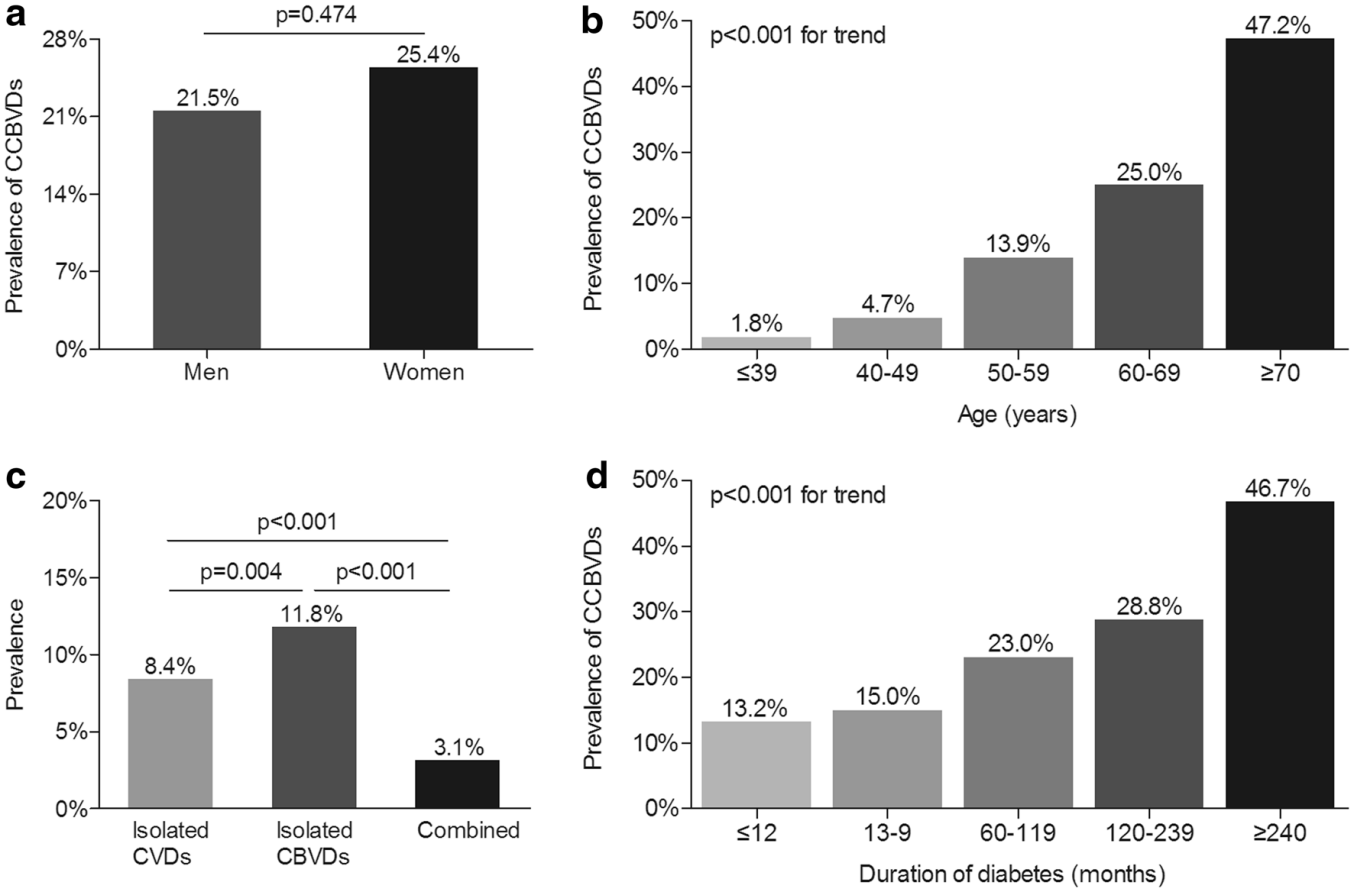
Characteristics of self-reported CCBVDs of the whole subjects **a** The prevalence of CCBVDs stratified by sex after adjusting on age and DD. The p value for sex comparison was 0.474. **b** The prevalence of CCBVDs stratified by age after adjusting on sex and DD. The p value for group comparison was <0.001. **c** The distribution of CCBVDs after adjusting on sex, age and DD. The prevalence of isolated CVDs, isolated CBVDs and both CVDs and CBVDs was successively 8.4, 11.8 and 3.1 %. The p value for group comparison was <0.001. **d** The prevalence of CCBVDs stratified by duration of diabetes after adjusting on sex and age. The p value for group comparison was <0.001

Characteristics of CCBVEs and self-reported CCBVDs stratified by sex, age and DD in all participants are shown in Figs. 1 and 2. Figures 1a and 2a demonstrated that there was no sex-related significant difference in the prevalence of CCBVEs and self-reported CCBVDs, respectively. However, a remarkable increase with age and DD was successively found in the prevalence of CCBVEs and CCBVDs among the whole patients (Figs. 1b, d, and  2b, d). Additionally, the prevalence of isolated CBVEs (12.5 %) was notably higher than that of isolated CVEs (2.1 %) and combined CVEs and CBVEs (0.9 %) in type 2 diabetes. The same trend was found for self-reported cardiovascular and cerebrovascular diseases (11.8 % for isolated CBVDs, 8.4 % for isolated CVDs, and 3.1 % for combined CVDs and CBVDs, respectively) in type 2 diabetes (Figs. 1c and 2c).

### Comparison of IMT in carotid and femoral among the three groups

**Fig. 3:**
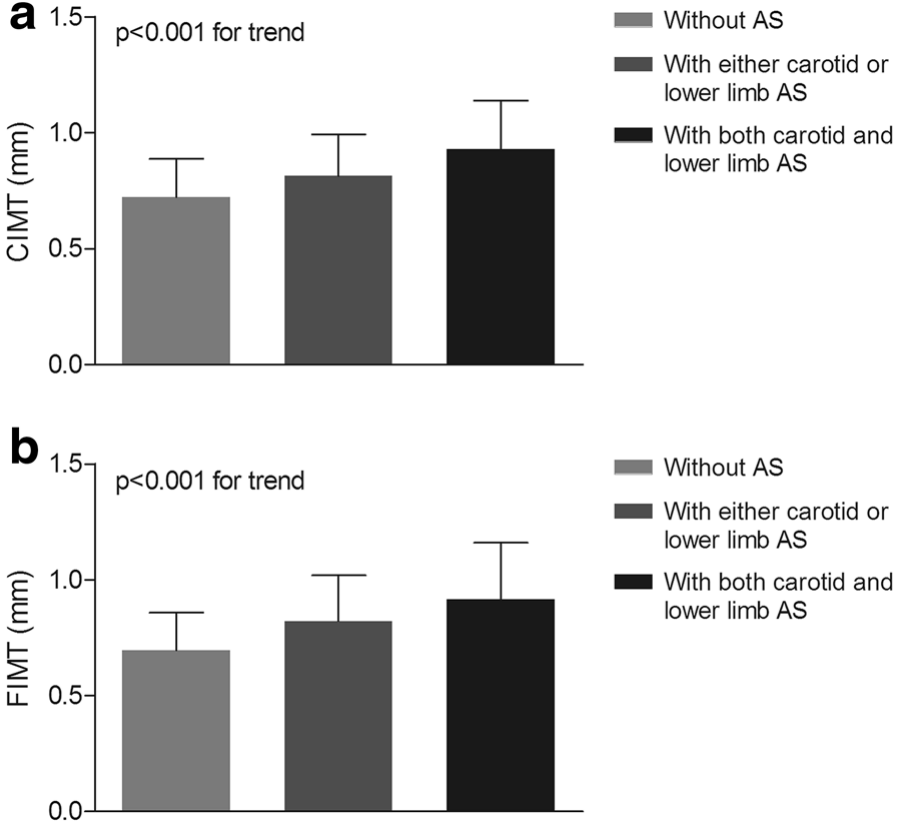
Comparison of IMT in carotid and femoral among the three groups **a** Comparison of CIMT among the three groups after adjustment on age, sex and DD. The p value for group comparison was <0.001. **b** Comparison of FIMT among the three groups after adjustment on age, sex and DD. The p value for group comparison was <0.001

After controlling for age, sex and DD, a comparison of IMT in carotid and femoral (CIMT and FIMT) among the three groups is shown in Fig. 3. Intriguingly, significantly higher value of CIMT was observed across the three groups (0.72 ± 0.16 mm, 0.82 ± 0.18 mm and 0.92 ± 0.21 mm successively for the subjects without atherosclerosis, those with either carotid or lower limb atherosclerosis and those with both carotid and lower limb atherosclerosis, p < 0.001 for trend) (Fig. 3a). The same trend was found for FIMT value among the three groups (0.70 ± 0.16 mm, 0.82 ± 0.20 mm and 0.91 ± 0.24 mm, respectively, p < 0.001 for trend) (Fig. 3b).

### Comparison of the prevalence of CCBVEs and self-reported CCBVDs among the three groups

**Fig. 4:**
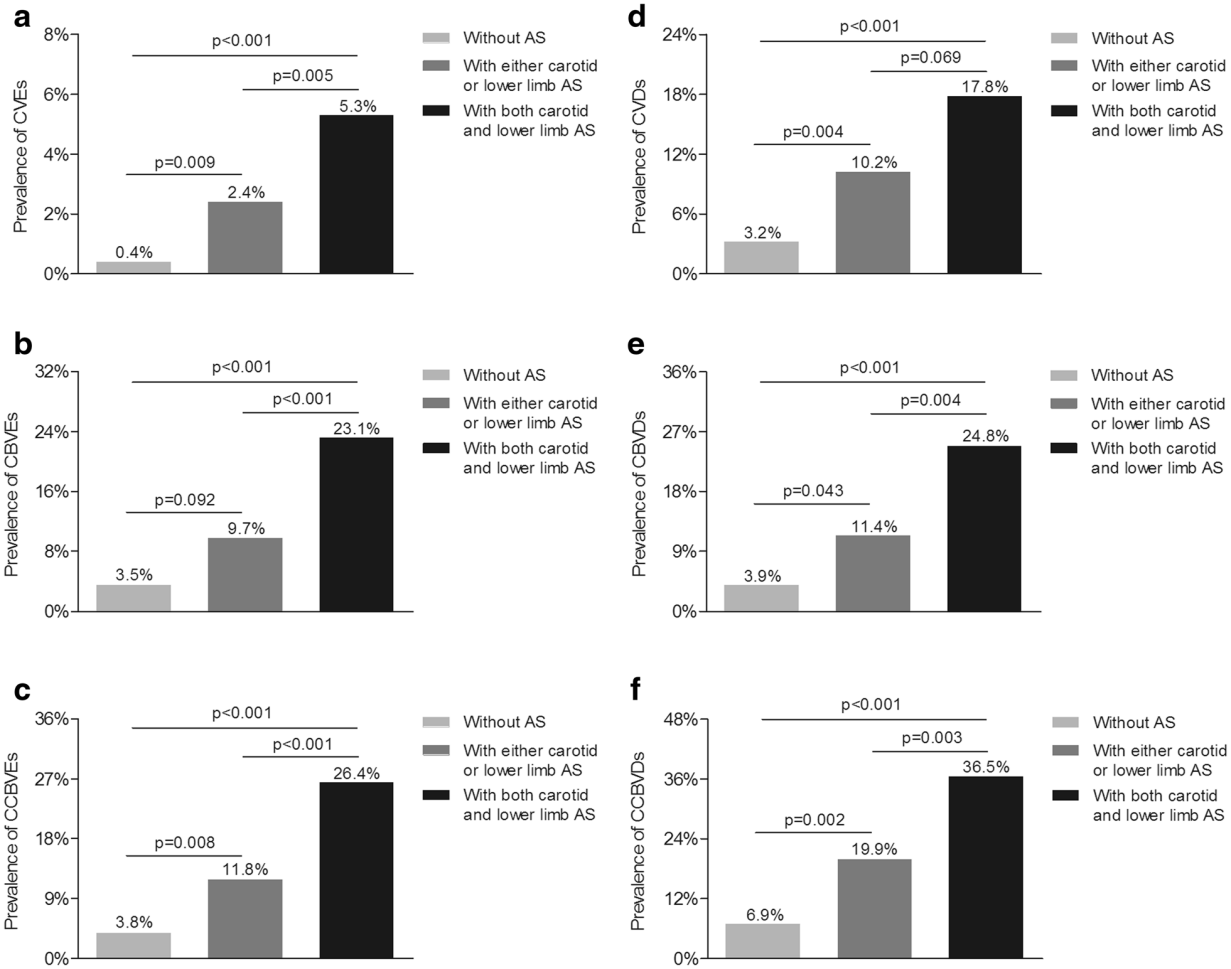
Comparison of the prevalence of CCBVEs and self-reported CCBVDs among the three groups **a** Comparison of the prevalence of CVEs among the three groups after adjustment on age, sex and DD. The p value for group comparison was <0.001. **b** Comparison of the prevalence of CBVEs among the three groups after adjustment on age, sex and DD. The p value for group comparison was <0.001. **c** Comparison of the prevalence of CCBVEs among the three groups after adjustment on age and sex. The p value for group comparison was <0.001. **d** Comparison of the prevalence of CVDs among the three groups after adjustment on age, sex and DD. The p value for group comparison was <0.001. **e** Comparison of the prevalence of CBVDs among the three groups after adjustment on age, sex and DD. The p value for group comparison was <0.001. **f** Comparison of the prevalence of CCBVDs among the three groups after adjustment on age, sex and DD. The p value for group comparison was <0.001

Figure 4 illustrates the comparison of the prevalence of CCBVEs and self-reported CCBVDs among the three groups. After adjusting for age, sex and DD, there was gradual rise in the prevalence of CVEs and self-reported CVDs from the group without atherosclerosis, the group with either carotid or lower-limb atherosclerosis, to the group with concomitant carotid and lower-limb atherosclerosis (p < 0.001 for trend) (Fig. 4a, d). Moreover, a remarkable increase in the prevalence of CBVEs and self-reported CBVDs was also observed across the three groups (p < 0.001 for trend) (Fig. 4b, e). Likewise, the prevalence of CCBVEs and self-reported CCBVDs also dramatically rose across the three groups (p < 0.001 for trend) after controlling for age, sex and DD (Fig. 4c, f).

### Associations between atherosclerosis and CCBVEs, self-reported CCBVDs

Table 2 presents the association between atherosclerosis and CCBVEs in type 2 diabetes. We found that the patients with either carotid or lower limb atherosclerosis and those with both carotid and lower limb atherosclerosis had 6.405- and 13.841-fold risk of CVEs,1.429- and 2.218-fold risk of CBVEs, and 1.690- and 2.708-fold risk of CCBVEs respectively, compared with the subjects without atherosclerosis after adjustment on age, sex, smoking, alcohol, DD, MetS, hypertension and CKD (Model I). Even after controlling on various clinical indicators (Model II and III), those with either carotid or lower limb atherosclerosis and those with the coexistence of carotid and lower extremity atherosclerosis still had 4.742- and 11.306-fold risk of CVEs, 1.389- and 2.092-fold risk of CBVEs, and 1.724- and 2.869-fold risk of CCBVEs, respectively.

**Table 2:** Associations between carotid, lower-limb atherosclerosis and CVEs, CBVEs, CCBVEs

	ORs (95 % CI)			p value for trend
	Without AS	With either carotid or lower limb AS	With both carotid and lower limb AS	
CVEs				
Model I	1 (ref)	6.405 (1.484–27.651)	13.841 (3.322–57.677)	<0.001
Model II	1 (ref)	6.090 (1.405–26.399)	13.302 (3.183–55.589)	<0.001
Model III	1 (ref)	4.742 (1.050–21.411)	11.306 (2.602–49.129)	<0.001
CBVEs				
Model I	1 (ref)	1.429 (0.869–2.350)	2.218 (1.351–3.642)	0.001
Model II	1 (ref)	1.401 (0.833–2.356)	2.169 (1.290–3.645)	0.002
Model III	1 (ref)	1.389 (0.780–2.475)	2.092 (1.1710–3.738)	0.011
CCBVEs				
Model I	1 (ref)	1.690 (1.052–2.714)	2.708 (1.681–4.362)	<0.001
Model II	1 (ref)	1.685 (1.030–2.756)	2.749 (1.675–4.510)	<0.001
Model III	1 (ref)	1.724 (1.001–2.966)	2.869 (1.660–4.960)	<0.001

Model I: Adjusted for age, sex, smoking, alcohol, DD, MetS, hypertension and CKD

Model II: Adjusted for age, sex, smoking, alcohol, DD, MetS, hypertension, CKD, BMI, WHR, SBP and DBP

Model III: Adjusted for age, sex, smoking, alcohol, DD, MetS, hypertension, CKD, BMI, WHR, SBP, DBP, FPG, 2h PPG, HbA1c, CRP, FCP, 2h C-P, SCr, SUA, TG, TC, HDL-C and LDL-C

Table 3 displays the association between atherosclerosis and CCBVDs in type 2 diabetes. After adjustment on age, sex, smoking, alcohol, DD, MetS, hypertension and CKD (Model I), the patients with either carotid or lower limb atherosclerosis and those with both carotid and lower limb atherosclerosis had 1.839- and 2.305-fold risk of CVDs, 1.515- and 2.123-fold risk of CBVDs, and 1.587- and 2.023-fold risk of CCBVDs respectively, relative to those without atherosclerosis. Even after adjusting clinical and biochemical parameters (Model II and III), the patients with either carotid or lower limb atherosclerosis and those with the coexistence of carotid and lower extremity atherosclerosis still had 1.899- and 2.667-fold risk of CVDs, 1.582- and 2.114-fold risk of CBVDs, and 1.705- and 2.147-fold risk of CCBVDs, respectively.

**Table 3:** Associations between carotid, lower-limb atherosclerosis and self-reported CVDs, CBVDs, CCBVDs

	ORs (95 % CI)			p value for trend
	Without AS	With either carotid or lower limb AS	With both carotid and lower limb AS	
CVDs				
Model I	1 (ref)	1.839 (1.118–3.025)	2.305 (1.387–3.832)	0.005
Model II	1 (ref)	2.002 (1.189–3.371)	2.543 (1.492–4.333)	0.002
Model III	1 (ref)	1.899 (1.057–3.413)	2.667 (1.467–4.847)	0.003
CBVDs				
Model I	1 (ref)	1.515 (0.949–2.418)	2.123 (1.327–3.397)	0.002
Model II	1 (ref)	1.472 (0.905–2.396)	2.077 (1.272–3.389)	0.004
Model III	1 (ref)	1.582 (0.920–2.721)	2.114 (1.220–3.661)	0.017
CCBVDs				
Model I	1 (ref)	1.587 (1.103–2.283)	2.023 (1.394–2.936)	0.001
Model II	1 (ref)	1.597 (1.093–2.333)	2.029 (1.376–2.993)	0.001
Model III	1 (ref)	1.705 (1.115–2.605)	2.147 (1.388–3.320)	0.002

Model I: Adjusted for age, sex, smoking, alcohol, DD, MetS, hypertension and CKD

Model II: Adjusted for age, sex, smoking, alcohol, DD, MetS, hypertension, CKD, BMI, WHR, SBP and DBP

Model III: Adjusted for age, sex, smoking, alcohol, DD, MetS, hypertension, CKD, BMI, WHR, SBP, DBP, FPG, 2h PPG, HbA1c, CRP, FCP, 2h C-P, SCr, SUA, TG, TC, HDL-C and LDL-C

## Discussion

Both carotid and femoral atherosclerosis has been supported as potential predictors of cardiovascular and cerebrovascular morbidity and mortality in different populations [15, 21, 22]. At present, most studies focused on the associations of carotid atherosclerosis with cardiovascular and cerebrovascular risks, but investigations about the relationships between the concomitant presence of carotid and lower extremity atherosclerosis and cardio-cerebrovascular risk were extremely scarce in patient with type 2 diabetes. Therefore, we carried out this cross-sectional study to assess the association between the concomitant presences of carotid and lower extremity atherosclerosis and CCBVEs, self-reported CCBVDs in Chinese hospitalized type 2 diabetics. Our results strongly indicated that patients with atherosclerosis had remarkably higher risk of CCBVEs and self-report CCBVDs than those without atherosclerosis even after adjustment for other cardiovascular risk factors. Most importantly, the coexistence of carotid and lower extremity atherosclerosis further increases the risk of CCBVEs and self-report CCBVDs in type 2 diabetes.

Consistent with other studies [35, 36], we also found the prevalence of CCBVEs and self-reported CCBVDs in Chinese type 2 diabetic patients successively increased with age and duration of diabetes. Several literatures reported that men had a higher risk of coronary heart disease whereas women had a greater propensity of developing stroke [37, 38]. On the contrary, we explored that there was no significant gender difference in the prevalence of CCBVEs and self-reported CCBVDs, as this can be mainly explained by the fact that CCBVEs and self-reported CCBVDs included both cardiovascular and cerebrovascular events/diseases in our study.

Similar to our previous and other study [30, 32, 39], subjects with atherosclerosis had significantly higher values of CIMT and FIMT than those without atherosclerosis. More importantly, we observed that CIMT and FIMT values were notably elevated in the patients with concomitant presence of carotid and lower extremity atherosclerosis than in those with either carotid or lower limb atherosclerosis, which suggested that CIMT and FIMT closely related to atherosclerosis severity.

Studies revealed that plaques in carotid, femoral arteries were strong predictors of CVEs and self-reported CVDs in a variety of populations, including cardiological patients, hypertensive patients and general subjects [40–43]. For example, Leng et al. [43] showed that subjects with femoral plaque had significantly 2.2-fold and 1.7-fold odds of previous ischemic heart disease and angina in an older British population. Also, the multi-ethnic study of atherosclerosis displayed carotid plaque independently predict cardiovascular events at 7.8-year mean follow-up and improve risk prediction for coronary heart disease when added to Framingham risk factors [44]. Consistent with these studies, our current study, including a sufficiently large number of cases, provides further support to the opinion that atherosclerosis positively and independently associated with cardiovascular risk in type 2 diabetes. In the current study, we not only found that patients with atherosclerosis had pronouncedly higher prevalence of CVEs and self-reported CVDs than those without atherosclerosis after controlling for age, sex, and duration of diabetes, but also explored that patients with atherosclerosis still had 4.742-fold for CVEs and 1.899-fold risk for self-reported CVDs after controlling for other potential confounders, compared with the patients without atherosclerosis.

Contrary to above results, some scholars have questioned the independent correlation between atherosclerosis and CVEs, self-reported CVDs [24, 25]. Adeseun and colleagues [24] found that they were unable to detect any statistical difference regarding carotid plaque as a predictor of self-reported prevalent cardiovascular disease in the chronic kidney disease population. Several reasons could explain the difference. Firstly, self-report of cardiovascular disease may introduce misclassification bias. Moreover, different population, different race, and different measure methods may also contribute to the discrepancy. For example, population in study of Adeseun et al. was comprised of participants with chronic kidney disease and a higher proportion of black [24], whereas our study population was consisted of diabetic patients largely with normal renal function and all from Asian.

More importantly, we found that the coexistence of carotid and lower extremity atherosclerosis further enhances the prevalence of both CVEs and self-reported CVDs, compared to either carotid or lower-limb atherosclerosis independent of the other atherosclerosis indicators. In accordance with our findings, Davidsson et al. [45] showed that 2.53-fold risk for cardiovascular events in concomitant presence of carotid and femoral plaques was higher than 2.09-fold in carotid plaques and 1.99-fold in femoral plaques in Sweden middle-aged men during 10 years of follow-up. Furthermore, compared with Davidson et al. study [45], the association between atherosclerosis and cardiovascular events in our study was probably more precisely evaluated by measuring “bilateral” carotid arteries and “bilateral” femoral artery, while Davidson et al. only measured carotid arteries and “one side” of femoral arteries. In addition, investigations regarding the relationship between the coexistence of carotid and lower-limb atherosclerosis and CVEs, self-reported CVDs remained scarce, top disputes were around the degree of relationship between carotid, femoral atherosclerosis and CVEs, self-reported CVDs. For example, some studies reported that the correlation between coronary and femoral artery atherosclerosis were slightly weaker as compared to the relationship between carotid and coronary arteries, although carotid atherosclerosis and lower limb atherosclerosis are strong predictors of cardiovascular diseases [46–48]. Contrary to this, Sosnowski et al. [49] found that the femoral atherosclerotic lesions were more informative than carotid atherosclerotic lesions in risk estimations for the extent and severity of coronary artery disease after controlling on traditional cardiovascular risk factors for 410 perform coronary angiography. The discordant results may be explained below: firstly, the Angina Prognosis Study [22] indicated that plaques in the carotid artery were related to cardiovascular death or non-fatal myocardial infarction, whereas plaques in the femoral artery were related to revascularization after adjustment for a wide variety of traditional cardiovascular risk factors. Moreover, different factors such as local rheological conditions, vessels’ structure and stiffness influenced atherosclerosis development in the carotid and femoral arteries [50–52], and different measure methods were also adopted in above studies. For example, lower-extremity atherosclerosis was reflected by the ankle-arm index in the Rotterdam Study while lower-limb ultrasound was used by us and Sosnowski et al. [49], which could contribute to the inconsistencies among these studies. Base on this, we applied the combination of carotid and lower-limb ultrasound to assess the risk of CVEs and self-reported CVDs. Our results strongly indicated that patients with the coexistence of carotid and lower extremity atherosclerosis had prominently higher risk for both CVEs and self-reported CVDs than those with either carotid or lower-limb atherosclerosis and those without atherosclerosis.

Currently, studies regarding the association with atherosclerosis and CBVEs, self-reported CBVDs mainly centered on the relationship of plaque morphologic abnormalities, such as irregular surface and hypoechoic characteristics, and CBVEs [53–55]. For example, Mathiesen et al. [56] showed that echolucent carotid plaques with a higher content of lipid and hemorrhage are associated with increased risk for CBVEs in subjects with carotid stenosis. Many studies have also demonstrated MRI, rather than B-mode ultrasound, is able to distinguish plaque tissue characteristics and even quantify plaque components [57–59]. However, in comparison with B-mode ultrasound, MRI also has its disadvantage like higher price, costs longer time, and not available for everyone especially persons with metallic foreign body or pacemaker, so carotid MRI merely used as patients with carotid diseases especially carotid stenosis at present [53, 60, 61]. Therefore, in our current study, we investigated the association with the presence of plaques and CBVEs, CBVDs via both carotid and lower limb artery ultrasounds. And our results demonstrated that plaque occurrence in either carotid or lower limb arteries was not significantly associated with an increased risk of both CBVEs and self-reported CBVDs, consistent with Leng et al. [43]. Most importantly, to our great surprise, patients with the coexistence of carotid and lower extremity atherosclerosis had 2.092-fold for CBVEs and 2.114-fold risk for self-reported CBVDs relative to the patients without atherosclerosis even after adjusting for various risk factors. Our above results intensely indicated that combined application of carotid and lower limb ultrasound examinations to determine plaque is helpful to risk evaluation and prediction of both CBVEs and self-reported CBVDs in patients with type 2 diabetes.

Finally, we comprehensively assessed the relation of atherosclerosis and CCBVEs, self-reported CCBVDs in patients with type 2 diabetes. Our study displayed that not only either carotid or lower limb atherosclerosis was obviously related to increase cardio-cerebrovascular risks in type 2 diabetes, but also the concomitant presence of carotid and lower extremity atherosclerosis can further increase cardio-cerebrovascular risks in patients with type 2 diabetes. The above results indicated that clinicians could carry out drug intervention to prevent CCBVEs, self-reported CCBVDs for patients by referring to plaque conditions in practice.

To the best of our knowledge, this is the first time to systematically investigate the relationship between the concomitant carotid and lower-limb atherosclerosis and CCBVEs, self-reported CCBVDs with a large sample in Chinese patients with type 2 diabetes. Our results strongly indicated that patients with the coexistence of carotid and lower extremity atherosclerosis had prominently higher risk for both CCBVEs and self-reported CCBVDs than those with either carotid or lower-limb atherosclerosis and those without atherosclerosis, which suggested that the combination with carotid and lower limb ultrasound should be a better method for the detection of atherosclerosis to predict the probability of CCBVEs and self-reported CCBVDs.

## Limitations

Our study has important clinical implications on early detection of cardiovascular and cerebrovascular risks in patients with type 2 diabetes. However, there were several limitations in our study. Firstly, our current study performed a single center cross-sectional study even after adjustment analysis, but some unmeasured hidden biases may still exist and thus we will do further research in our prospective studies to verify above results. Secondly, patients in our study were hospitalized type 2 diabetes, and thus our current results may not be available to the general. Thirdly, CVDs, CBVDs and CCBVDs were mainly relied on the patient’s self-report, which may more or less introduce misclassification bias, so we strictly defined CVEs, CBVEs and CCBVEs in our study. Fourthly, we did not evaluate the impact of medical intervention on CCBVEs and CCBVDs.

## Conclusions

Either carotid or lower limb atherosclerosis was obviously related to increased cardiovascular and cerebrovascular risks in type 2 diabetes. The concomitant presence of carotid and lower extremity atherosclerosis further increases cardiovascular and cerebrovascular risks in patients with type 2 diabetes. Therefore, in clinical practice, combined application of carotid and lower extremity arterial ultrasound should be advocated to identify those subjects with high cardiovascular and cerebrovascular risks.

## Abbreviations

CVEs: isolated cardiovascular events
CBVEs: isolated cerebrovascular events
CCBVEs: cardio-cerebrovascular events
CDs: isolated cardiovascular diseases
CVDs: isolated cerebrovascular diseases
CCBVDs: cardio-cerebrovascular diseases
CIMT: carotid intima-media-thickness
DD: duration of diabetes
CKD: chronic kidney disease
Mets: metabolic syndrome
BMI: body mass index
WHR: waist-to-hip ratio
SBP: systolic blood pressure
DBP: diastolic blood pressure
TC: total cholesterol
TTG: total triglycerides
HDL-C: high-density lipoprotein cholesterol
LDL-C: low-density lipoprotein cholesterol
HbA1c: glycated hemoglobin a1c
FPG: fasting plasma glucose
2-h PPG: 2-h postprandial plasma glucose
FCP: fasting C-peptide
2-h PCP: 2-h postprandial C- peptide
ALT: alanine aminotransferase
Cr: creatinine
UAE: urinary albumin excretion
eGFR: estimated glomerular filtration rate
SUA: serum uric acid
CRP: C-reactive protein
IIAs: insulin or insulin analogues
LLDs: lipid-lowering drugs
AHAs: antihypertensive agents
APAs: anti-platelet agents
